# Clinical Assessment of Introducing Locoregional Anaesthesia Techniques as Part as the Intraoperative Analgesia Management for Canine Ovariohysterectomy in a Veterinary Teaching Hospital

**DOI:** 10.3390/ani12151939

**Published:** 2022-07-29

**Authors:** Jaime Viscasillas, Ariel Cañón, Eva Hernández, Agustín Martínez, Reyes Marti-Scharfhausen, Pilar Lafuente, José Ignacio Redondo

**Affiliations:** 1 Departamento Medicina y Cirugía Animal, Facultad de Veterinaria, Universidad Cardenal Herrera-CEU, CEU Universities, 46115 Valencia, Spain; arielcanonp@gmail.com (A.C.); eva.hernandezmagana@uchceu.es (E.H.); agustin.martinez.vet@gmail.com (A.M.); reyesvet92@gmail.com (R.M.-S.); nacho@uchceu.es (J.I.R.); 2 Ciencias de la Salud, UNIR-Universidad Intenacional de La Rioja, 26006 Logroño, Spain; mp_lafuente@yahoo.es

**Keywords:** quadratus lumborum block, TAP block, epidural, analgesia, ovariohysterectomy

## Abstract

**Simple Summary:**

Many analgesic protocols are described to manage pain during ovariohysterectomy. Over the past few years, loco-regional anaesthesia techniques have been introduced in veterinary anaesthesia. Therefore, students should be aware of these techniques and trained on them. This retrospective study assessed four different methods of providing analgesia for ovariohysterectomy (i.e., epidural analgesia (EPIDURAL), quadratus lumborum block (QLB), transversus abdominis plane (TAP) block, or systemic analgesia (GENERAL). Undergraduate students performed all the loco-regional anaesthesia procedures under the direct supervision of a qualified anaesthetist. Considering the inclusion criteria, 120 dogs were included in this study (22 EPIDURAL, 32 QLB, 39 TAP, 27 GENERAL). Based on our study results, we can conclude that all these four analgesic protocols are suitable for controlling nociception during ovariohysterectomy. All of them required a similar number of analgesic rescues and showed a very low rate of complications. No matter the technique, loco-regional anaesthesia showed better quality of recovery than just systemic analgesia. A significatively lower dose of methadone was used for premedication in the loco-regional anaesthesia groups compared with the systemic analgesia group. Epidural analgesia was the only technique in which the concentration of the volatile agent was lower than in the other groups. No complications related to loco-regional anaesthesia techniques were recorded; therefore, this indicates that it would be safe for students to perform when the procedure is conducted under direct supervision.

**Abstract:**

This study compared four methods to provide intraoperative analgesia during canine ovariohysterectomy in a veterinary teaching hospital. A retrospective study was designed to assess the nociceptive response, cardiorespiratory stability, quality of recovery and complications of four analgesic protocols: epidural analgesia (EPIDURAL group), Quadratus Lumborum block (QLB group), Transversus Abdominis Plane block (TAP group), and just systemic analgesia (GENERAL group). Undergraduate students carried out all the loco-regional techniques under the direct supervision of a qualified anaesthetist. A total of 120 cases met the inclusion criteria and were included in the study and were distributed as follows: 22, 27, 32 and 39 cases with EPIDURAL, GENERAL, QLB and TAP groups, respectively. Data were analysed with statistical software R using different statistical methods. Significant differences among groups were defined as *p* < 0.05. Based on our results, all the groups needed the same number of rescue analgesia during the intra-operative period. The use of loco-regional techniques anticipated a better quality of recovery compared with the general group. The EPIDURAL group showed a statistically lower expired fraction of sevoflurane. No differences were found regarding complications. In conclusion, these four analgesic methods are suitable and safe to be performed for canine ovariohysterectomy, although loco-regional techniques might have some advantages.

## 1. Introduction

Ovariohysterectomy is a very common surgical procedure in veterinary medicine [1]. In most of the cases, this surgery is elective, and animals are young and healthy. Therefore, it is also a surgical procedure used in veterinary teaching hospitals to teach students how to carry out not only the surgical procedure but also the anaesthetic management. Ovariohysterectomy has been also used as a model for somatic and visceral pain since both components are involved during the surgery [2,3,4,5]. The abdominal wall, at the level of the surgical incision for ovariohysterectomy, is innervated by lumbar nerve roots [6] and ovaries and uterus for the autonomic system. Therefore, it is a good surgical procedure to assess both the somatic and visceral analgesia in healthy animals without comorbidities. 

Many analgesic options have been described in the veterinary literature to manage intraoperative nociception during this surgery [7]. Systemic analgesics (such as opioids, ketamine, alpha_2_ agonists, NSAIDs, etc.) are widely used [7,8,9]. However, over the last years, many locoregional anaesthesia techniques have been used together with systemic analgesia to obtain a multimodal approach to nociception and decrease the dose and the potential side effects of systemic analgesics and anaesthetic drugs [10,11,12]. Some locoregional anaesthetic techniques such as infiltration or splash block are very easy to be carried out and have shown good results in previous studies in small animals [12]. Other locoregional anaesthetic techniques are more advanced and need a learning curve to reach a good success rate [13]. This is the case of techniques such as epidural, transversus abdominis plane (TAP) block or quadratus lumborum block (QLB). All these three techniques have been found as good analgesic options in previous studies on small animals. All of them have proved to decrease other analgesic and anaesthetic drugs, decreasing their potential side effects and improving the quality of recovery [10,11,14]. However, as it was mentioned before, these techniques require more specific anatomical knowledge and clinical skill because ultrasonography is needed in at least two of them (TAP and QL block) [15,16]. Furthermore, they can cause serious complications such as neurological damage, bleeding and hypotension [17,18,19,20]. For this reason, it is mandatory to teach these techniques properly.

The objective of this study was to evaluate the clinical effect of introducing epidural, TAP, and QLB performed by students in a veterinary teaching hospital and compare its performance with systemic analgesia in dogs undergoing ovariohysterectomy. Therefore, we designed this retrospective observational study, hypothesising that, compared to systemic analgesia, loco-regional techniques:

(1) would reduce the number of dogs requiring intraoperative interventions to control nociception,
(2) would improve hemodynamic equilibrium during surgery,
(3) would improve the quality of recovery.

## 2. Materials and Methods

This retrospective study was conducted at the Veterinary Teaching Hospital of the Veterinary School CEU-Cardenal Herrera. The medical records of dogs undergoing ovariohysterectomy from February 2020 to January 2022 were included in this study. The inclusion criteria were:

(1) Dogs underwent ovariohysterectomy with one of the following methods to provide analgesia: epidural technique performed just with local anaesthetics (EPIDURAL group), quadratus lumborum block (QLB group), transverse abdominis plane block (TAP group) or just systemic analgesia (GENERAL group).
(2) Only mid-line laparotomy surgical approach.
(3) Dogs with status ASA I and II. Dogs which underwent ovariohysterectomy together with other surgical procedures or previous abdominal surgeries were excluded from the study.

Data collected were age, weight, type of analgesic protocol (EPIDURAL, QLB, TAP or GENERAL), opioid dose for premedication, use of alpha_2_ adrenergic agonist drugs for premedication, use of Non-Steroidal Anti-inflammatory Drugs (NSAIDs) before surgery, expired fraction of sevoflurane (FeSevo), temperature at the end of the surgery (T), heart rate (HR), respiratory rate (RR), mean arterial pressure (MAP), systolic arterial pressure (SAP), diastolic arterial pressure (DAP), use of rescue analgesia (defined as any bolus of an analgesic drug administered during surgery), hypotension (defined as mean arterial pressure less than 60 mmHg for more than 5 min), hypertension (defined as systolic blood pressure higher than 180 mmHg for more than 5 min), bradycardia (defined as heart rate lower than 60 beats per minute), tachycardia (defined as heart rate higher of 120 beats per minute) and quality of recovery (1. Good: quiet and maintain sternal in less than 5 min after extubation; 2. Fair: quiet and maintain sternal recumbency in less than 10 min after extubation; 3. Bad: agitated and need to administer either sedative or analgesic drugs). Finally, any complications recorded in the anaesthetic record such as bleeding, neurological side effects after recovery or long recovery were also described.

The statistical study was carried out using a statistical software (R Core Team, 2022, version 4.2.1, https://www.R-project.org, accessed on 1 March 2022, Vienna, Austria). The effect size was calculated with the pwr.2p.test function from the pwr package for groups of different sample sizes. The alpha error was set at 0.05, the beta error was 0.8, and the alternative hypothesis was considered “less than.” An effect size of −0.78 was obtained. 

The normality of the variables was verified with a Shapiro–Wilk test. The normality was studied using the Levene test. Neither of the variables met normality and homoscedasticity criteria. Because of this a robust statistical approach and a general linear model were chosen for comparing groups. 

The number of intraoperative analgesic rescues (Rescue), the quality of the recovery (Recovery) or events (Bradycardia, Tachycardia, Hypotension, Hypertension) were compared between groups using the chi-square test or the Fisher exact test when appropriate.

Comparison between groups of the variables heart rate (HR), respiratory rate (RR), temperature (T), mean arterial pressure (MAP), pulsoximetry value (SpO_2_), end-tidal carbon dioxide (EtCO_2_), inspiratory fraction of sevoflurane (FiSEV) and expiratory fraction of sevoflurane (FeSEV) over the time was performed using t1way function for independent samples, which computes a one-way ANOVA on trimmed means. In this analysis, either normality or homoscedasticity assumptions are not required. It uses a generalization of Welch’s method. Corresponding post hoc tests are in the lincon function. Statistical differences were considered if *p* < 0.05. The trim level for the means was 0.2. The data are presented numerically as the medians (minimum to maximum), confidence intervals 95% (CI 95%) and are graphically presented as the medians, interquartile ranges, and minimums and maximums.

## 3. Results

A total of 120 cases met the criteria and were distributed as follows: 22, 27, 32 and 39 cases in the EPIDURAL, GENERAL, QLB and TAP groups, respectively. Demographic data are presented in Table 1. 

No statistical differences were found regarding the use of opioids, alpha_2_ adrenergic agonist and NSAIDs in the premedication among the groups. The dose of methadone in the premedication was statistically lower in the locoregional analgesia groups compared with the systemic analgesia group (0.16 ± 0.5 versus 0.34 ± 0.6, *p* = 0.0121) (Table 2). 

The percentage of dogs requiring intraoperative interventions to control nociception was 18.2%, 31.3%, 28.2% and 25.9% in Epidural, QLB, TAP and GENERAL groups, respectively. No statistical differences were found among the groups (Table 3).

RECOVERY was better using locoregional techniques groups than in systemic analgesia (Table 4).

**Figure 1:**
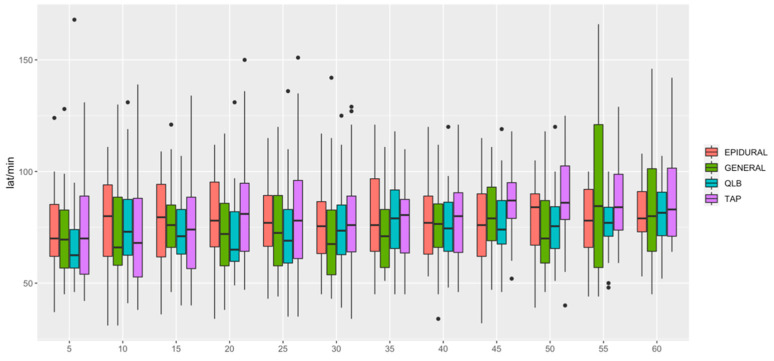
Graphic showing the different values of heart rate (HR) over time. A significant difference was found between General vs QLB and General vs TAP.

**Figure 2:**
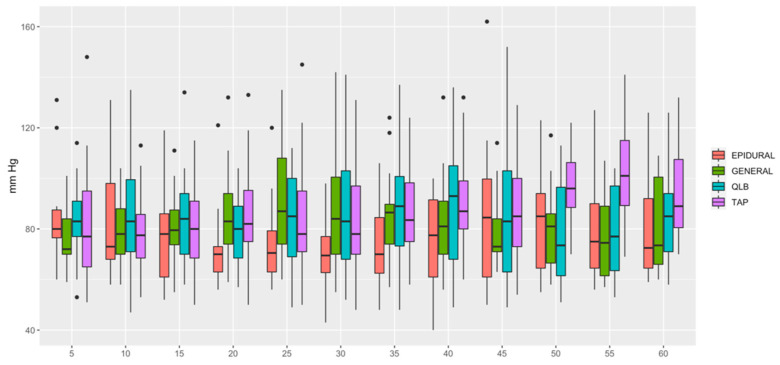
Graphic showing the different values of mean arterial pressure (MAP) over time. EPIDURAL group showed a significatively lower MAP than the rest of the groups.

**Figure 3:**
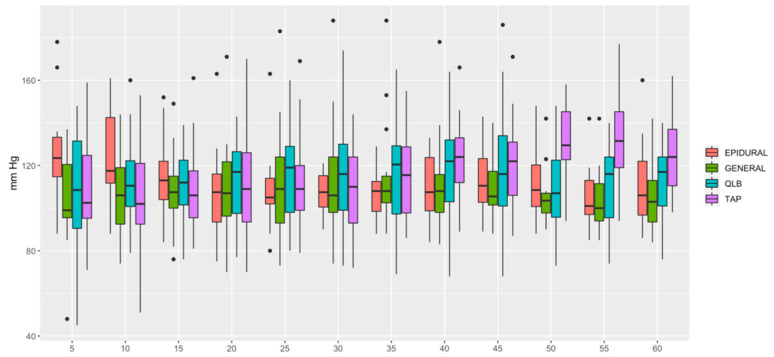
Graphic showing the different values of systolic arterial pressure (SAP) over time.

**Figure 4:**
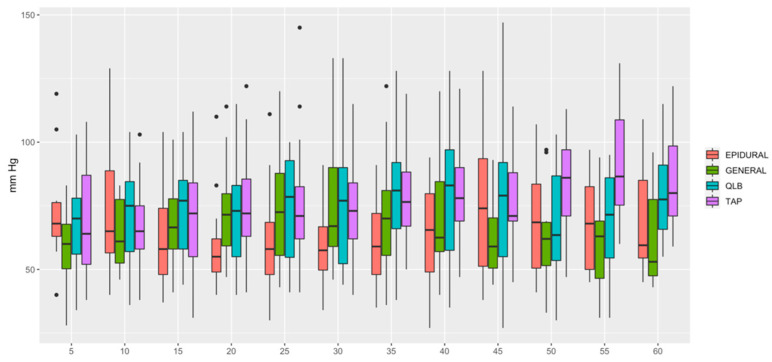
Graphic showing the different values of diastolic arterial pressure (DAP) over time.

HR (Figure 1) was lower in QLB than in TAP (*p* = 0.006) and lower in GENERAL than in TAP (*p* = 0.0349). MAP (Figure 2) was lower in EPIDURAL than in QLB (*p* = 0.00067), TAP (*p* < 0.0001) or GENERAL (*p* = 0.01777). SAP (Figure 3) was lower in GENERAL than in QLB (*p* = 0.0105) or TAP (*p* = 0.0032); SAP was lower in EPIDURAL than in TAP (*p* = 0.0389). DAP (Figure 4) was lower in EPIDURAL than in QLB (*p* = 0.00003) or TAP (*p* < 0.000001); DAP was lower in GENERAL than in QLB (*p* = 0.00381) or TAP (*p* = 0.00003). 

The prevalence of cardiovascular complications was similar among the groups (Table 5).

**Figure 5:**
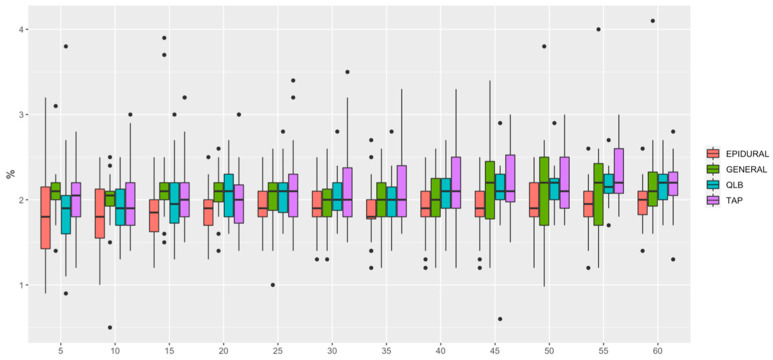
Graphic showing the different values of expired fraction of sevoflurane (FeSev) over the time. Epidural group showed a statistically lower FeSev than the rest of the groups.

FeSEV (Figure 5) was lower in EPIDURAL than in GENERAL (*p* = 0.00001), QLB (*p* = 0.00067) or TAP (0.00021). 

The rest of the studied parameters showed no statistical difference. No specific complications regarding locoregional anaesthesia techniques were recorded in the files.

## 4. Discussion

The present study shows that all analgesic protocols tested are similar regarding controlling the nociceptive response during ovariohysterectomy. The percentage of rescue analgesia ranged from 18.2% to 31.3% in EPIDURAL and QLB, respectively. However, the Epidural group had significatively fewer rescue analgesics than QLB. This result is contrary to a human study [21] in which patients receiving quadratus lumborum block for nephrectomies had reduced opioid requirements, reduced pain scores, and improved side effects relative to other analgesic modalities such as epidurals. 

Epidural analgesia success relies on the correct positioning of the tip of the needle inside the epidural space and adequate spreading of the local anaesthetic. Instead, QLB success needs two correct injections at the correct point and adequate spreading of the local anaesthetic. Furthermore, based on cadaveric studies performed in canine cadavers, there is still no clear answer about which approach and drug volume are the best. The fact that students carried out all the locoregional anaesthetic techniques could be why we found a different success rate between both techniques. Authors believe that the epidural technique´s learning curve can be steeper than QLB since just one injection is needed. Furthermore, several recognised methods (such as hanging drop, ultrasonography, neurostimulation, etc.) have been described [22] to assess the correct position of the tip of the needle in the epidural space.

In our study, the dogs treated with loco-regional anaesthesia received a significantly lower dose of methadone in the premedication. This finding could be related to the clinical experience of the anaesthetists in our institution. A low dose of opioids is usually given to animals receiving any loco-regional anaesthesia technique. Additionally, considering the initial dose of methadone administered to each group in the premedication, all the loco-regional anaesthesia groups had less opioid consumption no matter whether they needed rescue analgesia or not. This fact could be one of the reasons why recovery was statistically better with the locoregional techniques group compared with the GENERAL group. Other veterinary studies have shown good recovery quality and even good postoperative analgesia when locoregional techniques have been added to the analgesic protocol. Adequate analgesia and a low dose of systemic analgesic/anaesthetics drugs have been linked to good quality of recovery. The QLB has been proved to provide excellent recovery and good postoperative analgesia in dogs undergoing ovariohysterectomy with an opioid-free analgesic protocol [10]. The TAP block has also shown a good analgesic effect for the post-operatory period in cats undergoing ovariohysterectomy [14]. Finally, epidural analgesia based on local analgesics has also been shown to provide good analgesia, decreasing the amount of other systemic analgesics [11].

In the epidural analgesia group, 4 out of 22 patients (18.2%) needed rescue analgesia during the surgery. The epidural technique is a well-recognised method to provide potent analgesia for abdominal surgery [11]. One injection inside the epidural space will allow the local anaesthetic to spread along it and block the nerve roots and the nerve fibres that form the autonomic system, providing somatic and visceral analgesia for abdominal procedures. Therefore, it is very likely that the technique was not carried out adequately in cases that needed rescue analgesia. The epidural technique success rate has been reported in dogs as 5.5% [23], 11.3% [24], and 15% [25].

In the same way, QLB showed a higher success rate (90%) in another clinical study [10] with dogs undergoing ovariohysterectomy. The fact that anaesthetists with experience in loco-regional anaesthesia carried out all the techniques in those studies could be why the success rate in the locoregional anaesthesia groups is much higher than in our study in some cases. In our study, all the techniques were carried out by students, although always supervised by an anaesthesiologist.

Furthermore, the QLB has been described by injecting the local anaesthetic in three different anatomical locations. Although the same injection point was used in the mentioned study with a high success rate [10], it was impossible to retrieve this information from our files, so different points could be used. The injection point and volume can significantly impact the spreading of the local anaesthetic, as described in human and veterinary studies [10,26,27,28,29].

Epidural analgesia allowed a statistically significant lower FeSEV than the rest of the groups. The rest of the techniques needed similar FeSEV. Therefore, no sparing effect was found in our clinical study. Since it is a retrospective study, the authors cannot be sure whether the FeSEV could be decreased more in the other groups which did not receive rescue analgesia. Mean arterial pressure was statistically lower in the EPIDURAL group than in the rest. Therefore, perhaps the anaesthetists in charge of the epidural cases were more concerned about the MAP and tried to decrease the concentration of the volatile agent as much as possible. Nevertheless, hypotension was found more commonly in the EPIDURAL and QLB groups compared with TAP and GENERAL groups (50% and 46.9% compared with 28.2% and 29.6%, respectively). These values are similar to other studies published in veterinary anaesthesia in which hypotension was found in 37.9% of the cases anaesthetised with systemic analgesia [30].

The rate of complications is statistically not significant in all the groups. Hypotension was found in all of them but, as mentioned before, the rate is similar to the previously published study on dogs [30]. Interestingly, the EPIDURAL and QLB groups showed a higher percentage than the rest. This fact could be related to the sympathetic blockade that both techniques can cause. On the other hand, one important finding is that no complications related to the technique were found in the EPIDURAL, TAP and QLB groups, apart from the failure of the locoregional technique. This fact is essential since students performed all the techniques under the direct supervision of the veterinary anaesthetist in charge of the case. Other loco-regional anaesthesia techniques have provided satisfactory intraoperative analgesia during ovariohysterectomy in bitches [31,32,33,34]. Techniques such as splash block, infiltration or ovarian pedicle block are more accessible to carry out and cheaper than the ones we described in our study. They do not need advanced equipment such as an ultrasound machine, skills to guide the needle and administer the local anaesthetic in the correct location or more anatomical knowledge. However, the reason for teaching our students these advanced loco-regional anaesthesia techniques is that they can be helpful for other surgeries apart from ovariohysterectomies.

Furthermore, the epidural technique penetrates inside the vertebral canal. Therefore, it might have potentially serious complications such as neurological problems. Our results support that these advanced techniques can be taught on the clinical floor to the students under direct supervision in a safe way. However, a learning curve is needed for each person to reach a success rate. Unfortunately, since the design of our study is retrospective, we could not assess the learning curve [13,35].

This study has some limitations which need to be addressed. First, this is a retrospective study, and some information cannot be completely accurate. Furthermore, we could not consider the experience of the students who performed the technique since that information was not in the anaesthetic record. Another important fact is that we do not have information about how confident the anaesthetist in charge of the case was regarding how well the student carried out the technique. Finally, although we assessed the quality of recovery based on the anaesthetic records, the postoperative analgesic effect was not evaluated.

## 5. Conclusions

Based on our results, all the methods assessed in our study are similar to control nociception during ovariohysterectomy in dogs and provide cardiovascular stability. The use of locoregional techniques decreases the total dose of methadone and anticipates a better quality of recovery than just systemic analgesia. Students can safely carry out these loco-regional anaesthesia techniques under direct supervision. Further clinical studies are needed to assess the same techniques by experienced anaesthetists.

## Figures and Tables

**Table 1:** Demographic data showing number and percentage of gender, age, weight and ASA status.

	EPIDURAL (n = 22)	GENERAL (n = 27)	QLB (n = 32)	TAP (n = 39)	Overall (n = 120)
**AGE**					
Median [Min, Max]	2.00 [0.500, 10.0]	2.00 [0.700, 13.0]	3.00 [0.800, 15.0]	1.50 [0.600, 11.0]	2.00 [0.500, 15.0]
Missing	0 (0%)	0 (0%)	0 (0%)	1 (2.6%)	1 (0.8%)
**WEIGHT**					
Median [Min, Max]	16.3 [3.00, 36.0]	14.2 [3.00, 46.0]	14.3 [2.80, 165]	15.0 [2.50, 44.0]	15.0 [2.50, 165]
**ASA status**					
1	19 (86.4%)	22 (81.5%)	23 (71.9%)	30 (76.9%)	94 (78.3%)
2	3 (13.6%)	5 (18.5%)	9 (28.1%)	9 (23.1%)	26 (21.7%)

**Table 2:** Results on the cases that received alpha2 adrenergic agonists, opioids and NSAIDs for premedication in each group. There were no significant differences on the use of these drugs among the groups.

	EPIDURAL (n = 22)	GENERAL (n = 27)	QLB (n = 32)	TAP (n = 39)	Overall (n = 120)
**ALPHA_2_**					
YES	22 (100%)	24 (88.9%)	31 (96.9%)	37 (94.9%)	114 (95.0%)
NO	0 (0%)	3 (11.1%)	1 (3.1%)	2 (5.1%)	6 (5.0%)
**OPIOIDS**					
NO	3 (13.6%)	4 (14.8%)	7 (21.9%)	10 (25.6%)	24 (20.0%)
YES	19 (86.4%)	23 (85.2%)	25 (78.1%)	29 (74.4%)	96 (80.0%)
**NSAIDs**					
NO	11 (50.0%)	17 (62.9%)	21 (65.6%)	24 (61.5%)	78 (65.0%)
YES	11 (50.0%)	10 (37.1%)	11 (34.4%)	15 (38.5%)	42 (35.0%)
**METHADONE** **DOSE**					
	0.15 ± 0.5	0.34 ± 0.6	0.16 ± 0.5	0.17 ± 0.5	

**Table 3:** Number and percentage of dogs in each of the four groups requiring intraoperative interventions to control nociception. No statistical differences were found among the groups; EPIDURAL vs. GENERAL (*p* = 0.73), EPIDURAL vs. QLB (*p* = 0.35), EPIDURAL vs. TAP (*p* = 0.54), General vs. QLB (*p* = 0.78), General vs. TAP (*p* = 1) and QLB vs. TAP (*p* = 0.8).

	EPIDURAL (n = 22)	GENERAL (n = 27)	QLB (n = 32)	TAP (n = 39)
NO	18 (81.8%)	20 (74.1%)	22 (68.8%)	28 (71.8%)
YES	4 (18.2%)	7 (25.9%)	10 (31.3%)	11 (28.2%)

**Table 4:** Number and percentage of animals showing good and fair recovery in the general group compared with the locoregional group (involving EPIDURAL, QLB and TAP). Significative difference was found between both groups (*p* = 0.01). No significative differences were found among the EPIDURAL vs. QLB (*p* = 0.97), EPIDURAL vs TAP (0.78) and QLB vs. TAP (*p* = 0.65).

	GENERAL (n = 27)	LOCOREGIONAL (n = 93)	Overall (n = 120)
Good	21 (77.8%)	89 (95.7%)	110 (91.7%)
Fair	6 (22.2%)	4 (4.3%)	10 (8.3%)

**Table 5:** Number and percentage of complications found in each group. No significant differences were found among the groups, although the total number of complications was very low. No complications were found related to the realization locoregional anaesthesia technique.

	EPIDURAL (n = 22)	GENERAL (n = 27)	QLB (n = 32)	TAP (n = 39)	Overall (n = 120)
**BRADYCARDIA**					
Yes	7 (31.8%)	10 (37.0%)	9 (28.1%)	12 (30.8%)	38 (31.7%)
No	15 (68.2%)	17 (63.0%)	23 (71.9%)	27 (69.2%)	82 (68.3%)
**TACHYCARDIA**					
Yes	0 (0%)	0 (0%)	0 (0%)	0 (0%)	0 (0%)
No	22 (100%)	27 (100%)	32 (100%)	39 (100%)	120 (100%)
**HYPOTENSION**					
Yes	11 (50%)	8 (29.6%)	15 (46.9%)	11 (28.2%)	45 (37.5%)
No	11 (50%)	19 (70.4%)	17 (53.1%)	28 (71.8%)	75 (62.5%)
**HYPERTENSION**					
Yes	0 (0%)	0 (0%)	0 (0%)	1 (2.6%)	1 (0.8%)
No	22 (100%)	27 (100%)	32 (100%)	38 (97.4%)	119 (99.2%)
**OTHER COMPLICATIONS**					
	None	None	None	None	None

## Data Availability

Data supporting the reported results can be sent to anyone interested by contacting the corresponding author.
